# Personality and Psychiatric Disorders in Women Affected by Polycystic Ovary Syndrome

**DOI:** 10.3389/fendo.2014.00185

**Published:** 2014-11-12

**Authors:** Elisabetta Scaruffi, Alessandra Gambineri, Stefania Cattaneo, Jenni Turra, Roberto Vettor, Roberto Mioni

**Affiliations:** ^1^Edo ed Elvo Tempia Valenta Foundation, Biella, Italy; ^2^Division of Endocrinology, Department of Internal Medicine, S. Orsola-Malpighi Hospital, University of Bologna, Bologna, Italy; ^3^Scuola Universitaria Interfacoltà in Scienze Motorie (SUISM), University of Turin, Turin, Italy; ^4^Clinical Medical 3, Department of Medicine, Hospital of Padua, University of Padua, Padua, Italy

**Keywords:** polycystic ovary syndrome, personality, Rorschach, MCMI-III, psychopathology, personality disorder, psychiatric disorder

## Abstract

**Background:** Polycystic ovary syndrome (PCOS) is the most prevalent endocrine disorder among fertile women. Studies show reduced quality of life, anxiety, depression, body dissatisfaction, eating disorder, and sexual dysfunction, but the etiology of these disturbs remains still debated. The aim of our study is to verify whether this hyperandrogenic syndrome characterizes a strong psycho(patho)logical personality.

**Method:** Sixty PCOS subjects (mean age 25.8 ± 4.7 years) were evaluated by anthropometric, metabolic, hormonal, clinical, and psychological parameters. After the certainty of the diagnosis of PCOS, the Rorschach test, according to Exner’s comprehensive system (CS) and the Millon Clinical Multiaxial Inventory-III (MCMI-III) were administered to each patient. The control group, on which the comparison was carried out, was composed by 40 healthy and aged compared women who were exclusively administered the Rorschach test according to CS.

**Results:** MCMI-III evidenced axis II DSM-IV personality disorders [4.1% schizoid, depressive, sadistic, negativistic (passive–aggressive), and masochistic, 6.1% avoiding, 12.2% dependent, 20.4% histrionic, 16.3% narcissistic, 2.0% obsessive–compulsive], and axis I DSM-IV psychiatric disorders: 10.2% anxiety, 2.0% somatoform disorder and bipolar disorder, 16.3% major depressive disorder. Finally, we found 44.9% delusional disorder and 4.1% thought disorder. Rorschach test’s results show 53.1% reduced coping abilities and social skills, 55.1% depression, 30.6% perceptual distortion and cognitive slippage, 24.5% constantly alert and worry, 8.1% at risk for suicide, and finally about 50% of our patients had chronic stress.

**Conclusion:** PCOS women have relevant personality and psychiatric disorders, when compared with normal subjects.

## Introduction

Polycystic ovary syndrome (PCOS) is a heterogeneous gynecological endocrine disorder, characterized by chronic anovulation, hyperandrogenism, and hyperinsulinism with insulin resistance, which affects about 6–10% of women in reproductive age (1–3) and profoundly affects the quality of life of these subjects (4, 5). In fact, this hyperandrogenic syndrome is frequently characterized by hirsutism, acne, alopecia, obesity, and irregular periods with infertility and it is often treated as cosmetic problem. The existence of a linkage between these latter clinical features and reduced quality of life was frequently suggested in several researches (6–8). Other studies evidenced how physical symptoms caused mental disorders, but depression and anxiety seem to be independent of obesity and infertility, the most frequent symptoms of PCOS (9, 10). Also, the relationship between androgen excess and mood remains still controversial because clinical and biochemical parameters of hyperandrogenism seem not directly cause of depression (11). Again, diagnosis of PCOS was related to negative feelings, such as frustration (67%), anxiety (16%), and sadness (10%), although the (self-reported) level of knowledge about the illness was high and the women were aware that PCOS can be treated and is not a deadly disease (12). Other authors reported reduced scores in the sub-categories of perception about health in general, physical performances, general behavior, and limitations in family activities due to the “girls’ disease” (12). At the same time, higher scores were reported in the rating of health conditions, during the diagnostic pathway and above all the treatment period, further supporting a dependent effect between clinical features and psychological aspects. In different manner, several data obtained in PCOS subjects reported that differences for the QoL are more related to the subjective perception of the illness’s seriousness rather than to the clinical seriousness of the illness itself. These data, therefore, suggest that a subjective feeling of discomfort might be totally unrelated to the clinical picture, although objectively recorded. Again, other psychological aspects, such as depression (8, 13), and/or anxiety (14, 15), have been emphasized in PCOS, but only few articles analyzed women’s personality structure (16). In women with PCOS, Sahingoz et al. (17) assessed a high prevalence of psychiatric and personality disorders, about 29.0 and 14.0%, respectively. They also reported 9.6% of mood and 26.0% of anxiety disorders, 13.7% of social phobia, 11.0% of generalized anxiety, and 12.3% of avoidant personality disorders, according to DSM-IV axis I and II Diagnostic Criteria, respectively. Again, Patients with borderline personality disorders, diagnosed by SCID for DSM-IV, about 30.0% was affected by PCOS (18). Personality disorders seem to be characterized by the tendency to exhibit a tenuous stability, or lack of resilience, under conditions of stress, by the inflexibility to adapt and by vicious cycles that repeats once again their pathology. Again, the interaction of psychosocial stressors and personality characteristics leads to the expression of psychological symptoms, that is, axis II and axis IV interact to produce axis I. Since no studies used Rorschach test or Millon Clinical Multiaxial Inventory to analyze the personality structure of subjects with PCOS, we believe it is useful to carefully study and develop the personality aspects related to this hyperandrogenic syndrome, as well as the psychopathological ones. Moreover, to minimize the influence of diagnostic and therapeutic pathways, we only studied young women, with comparable social and school conditions, undergoing for the first time to the endocrine evaluation for clinical features of hyperandrogenism and/or disorders of menstrual cycle.

This study was performed in collaboration with the Division of Endocrinology, Department of Internal Medicine of St. Orsola-Malpighi Hospital, University of Bologna, and with the Clinical Medical 3 of Health Centre/Hospital-University of Padua, on the white patients coming from the northern provinces of Italy.

## Patients and Methods

### Subjects recruitment

Sixty Caucasian women, all of Italian origin, affected by certain diagnosis of PCOS, were enrolled to participate in this research study: 30 subjects from the Endocrinology Unit of St. Orsola-Malpighi Hospital – University of Bologna and 30 subjects from the Clinical Medical 3 of Health Centre/Hospital-University of Padua. For all patients, it was the first time they turned to a Public Health Institution to resolve their hyperandrogenic symptoms.

All women were hyperandrogenic (clinical presence of hirsutism with Ferriman–Gallwey score >8, acne or alopecia and/or elevated androgen levels: testosterone (*T*) > 2.1 nmol/L and or androstenedione (A) > 10.4 nmol/L or Free Androgen Index > 5 [FAI = Testosterone(nmol/L)/SHBG(nmol/L) × 100] and met the diagnosis of PCOS, according to the Rotterdam criteria (19). Ovarian ultrasound findings were performed by transvaginal or transrectal pelvic ultrasound (US ESAOTE F.U.5, Probe of 6.5 MHz, Milan, Italy) and PCO diagnosis were considered according to previous criteria (20). All subjects were no-smokers, no dieting, had a normal glucose tolerance test and performed normal physical activities, none drank alcoholic beverages or took any continuous medications. According to PCOS diagnostic criteria, none had thyroid dysfunction, hyperprolactinemia, type 2 diabetes mellitus or concomitant cardiovascular, renal, and liver dysfunctions. Other causes of hyperandrogenism, such as Cushing syndrome/disease and congenital adrenal hyperplasia, were excluded, after nocturnal fasting, by basal and/or stimulated (0.25 mg Synacthen^®^ iv, Biofutura Pharma S.p.A., Pomezia, Rome) plasma 17-OH-progesterone levels. At the beginning of the study, the insulin resistance and hyperinsulinism were, respectively, evaluated by using the homeostatic model assessment (HOMA-ir) [(fasting glucose (mmol/L) × fasting insulin (mIU/L)/22.5; n.v. <1.9 in our control group of at least 200 normal healthy subjects (data not shown)] and the 3-h oral glucose tolerance test (OGTT; 75 g of glucose) for plasma glucose, insulin, and C-peptide levels. Whole-body insulin sensitivity was calculated by using the insulin sensitivity index derived from the OGTT (ISI_COMPOSITE_ n.v. >6.0) according to Matsuda and DeFronzo (21) considering normo-sensitive subjects with values higher than 6.0 and low-sensitive those with values lower than 4.0, respectively.

All women were studied in the follicular phase (days 1–7) of the cycle, or after 2 or more months of amenorrhea, after a negative pregnancy test. After this diagnostic pathway, but before of any pharmacological treatment, at each subject was administered the Millon Clinical Multiaxial Inventory-III (MCMI-III) (22). The MCMI-III has been adapted to the Italian population by a research group, which has involves the Universities of Milan-Bicocca, Rome-La Sapienza, and Padua, coordinated by Prof. Alessandro Zennaro, University of Turin, Italy (23, 24). We used Millon words to explain personality disorder and clinical syndrome.

In a subsequent day, for the first time in subject affected by PCOS, Rorschach, which is considered a scientific, valid, and reliable projective test to assess personality, was administered according to the Exner’s comprehensive system (CS), once adapted to Italian population (24). The protocol was approved by the Local Ethics Committee of both Padua and Bologna Hospital, and all women gave their informed consent approval to collaborate with this research. Eleven women invalidated the Rorschach protocols because they have not given a sufficient number of answers in the first tables, so the original group of the patients was reduced to 49 patients. All subjects aged from 19 to a maximum of 39 years (25.80 ± 4.27 years), with comparable social and school degree (from 13 to 18 years of school). Forty five women, with age (from 19 to 41 years; 25.2 ± 6.0), social, and school degree comparable, were selected from a total of 2.500 records present in our database, and considered as control group. Each control subject had a normal menstrual cycle, was not hirsute, not taking any drugs or abusing alcohol, did not smoke, and was matched to the subjects with PCOS for body weight and age. Anonymity was assured to each patient and names were substituted by an alphanumerical progression.

### Tests and selection of variables

The tests chosen for the personality analysis of the sample were (a) the *Rorschach Test* administered, scored, and interpreted according to the Exner’s CS (23, 24); and (b) The MCMI-III (22) is composed by 175 true/false items with 28 scales of which 14 refer to personality disorders and 10 refer to clinical syndromes and the remaining 4 refer to test validity indices. In MCMI-III scoring system vary from 1 to 115 with a median score of 60 base rate (BR), scores of 85 or above evidence a personality disorder, according to DSM-IV axis II classification and/or the prominence of a clinical syndrome, according to DSM-IV axis I diagnosis (24, 25). Italian version of MCMI-III shows good internal validity (Cronbach’s alpha range for subscales = 0.66–0.90) and test-retest reliability (*r* = 0.84–0.96) (23). The Rorschach tables were administered, scored, and interpreted according to the Exner’s CS (24, 26, 27). It was decided to select, besides the 27 variables proposed by Exner and Andronikof-Sanglade (28) and by Bihlar and Carlsson (29), further variables that we consider useful FD, SumT, SumC′, SumV, SumY, Sum Shading, Sum Color Shading Blends, Sum Shading Shading Blends, Wsum6, and An + Xy. Taking a cue from the work of Sultan et al. (30), conducted on patients with mellitus diabetes (IDDM), we wished to also consider part of their proposed indices.

### Laboratory and plasma assays

Glucose was measured by using the glucose oxidase method (Gluco-Quant^®^; Roche Diagnostics GmbH, Mannheim Germany), its detection limit was 0.11 mmol/L. Insulin by means of the two-site chemiluminescent immunometric immunoassay (Immulite 2000; Diagnostic Products Corporation, Los Angeles, CA, USA) and its analytic sensibility was 2.0 μIU/mL. The intra- and inter-assay coefficients of variation (CVs) were <5.5 and 7.3%, respectively. LH, FSH, and E2 were measured by a competitive immunoassay with the use of an electrochemiluminescence immunoassay (ECLIA; Roche Diagnostics GmbH, Mannheim, Germany). Analytic sensibility of LH and FSH was 0.2 and 0.34 UI/L, respectively, whereas for E2 was 0.05 pmol/L. The intra- and inter-assay CVs were 1.8 and 5.2% for LH, 2.0 and 5.3% for FSH, 5.7 and 6.2% for E2, respectively. Testosterone, DHEA-s, 17-OHP, and A, after celite column chromatographic separation, were assayed as previously described (31). Serum T and DHEA-s were further measured by competitive immunometric chemiluminescent enzyme immunoassay (Immulite 2000; Diagnostic Products Corporation, Los Angeles, CA, USA). The detection limit was 0.35 nmol/L for T and 0.08 μmol/L for DHEA-s, respectively, the inter- and intra-assay CVs were below 7.4% for T and below 6% for DHEA-s. A and OHP were measured by using an enzyme-linked immunosorbent assay, ELISA (DRG Instruments GmbH, Marburg, Germany) and their detection limit was 0.6 nmol/L for A and 0.18 nmol/L for OHP, respectively, with intra- and inter-assay CVs <9.1 and 12.1% for A and 7.8 and 9.4% for OHP, respectively. SHBG was measured by using two-site chemiluminescent immunometric assay (Immulite 2000; Diagnostic Products Corporation, Los Angeles, CA, USA), its analytic sensibility was 0.8 nmol/L with intra- and inter-assay CVs <5.3 and 6.6%, respectively.

### Analysis of the data

The anthropometric and hormonal data were reported as mean ± SD. All continuous variables were compared using the Student’s *t*-test for unpaired data. Glycometabolic and hormonal data from PCOS and control groups were compared and analyzed with repeated measures by ANOVA. Mann–Whitney *U* test and Wilcoxon paired rank test were also used for evaluating the distribution of non-parametric values. Values for *p* < 0.05 were considered significantly different.

For an in-depth approach to the psychological investigation of our samples, we carried out the following analyses – comparison of the BR points obtained from the administration of MCMI-III under the original norms proposed by Millon et al. (22); statistical comparison of the average values of the Rorschach variables selected with the most recent norms proposed by Exner and Erldberg (26) through the use of confidence intervals (CI 95%); statistical percentage comparison of the threshold values of the same variables with those proposed by Exner (25), ambitent subject, non-patient norms. The reference to the descriptive statistics of the ambitent subjects is justified by the confirmation that, on average, this coping style is representative of the M: WSumC relationship present in the PCOS sample (*M* = 2.25: SumC = 4.133). Comparison of Rorschach Indices [suicide constellation (S-Con), coping deficit index (CDI), depression index (DEP-I), perceptual-thinking index (PTI), schizophrenia index (SCZI), hypervigilance index (HVI)] obtained from subjects with PCOS were done with those obtained from control group recorded in our database.

## Results and Discussion

Auxological and endocrine–metabolic parameters of our subjects are summarized in Table 1. As expected, hyperandrogenic parameters were elevated in women affected by PCOS and significantly different when compared with Control group. In particular, LH/FSH ratio and serum androgens, above all testosterone, were significantly higher in PCOS than in Controls. Also, metabolic parameters and insulin resistance were significantly elevated in patients with PCOS, whereas, as expected by the enrollment criteria, body weight (calculated as BMI) did not differ between the groups.

**Table 1:** **Characteristics of women with PCOS and healthy controls**.

	PCOS (*n* = 49)	Control (*n* = 45)	*p*
AGE (years)	25.8 ± 4.7	25.2 ± 5.9	n.s
BMI (kg/m^2^)	27.6 ± 4.8	24.9 ± 4.4	n.s
Waist (cm)	78.2 ± 6.8	69.5 ± 3.7	<0.05
PRL (μg/L)	23.0 ± 8.6	11.9 ± 5.3	<0.05
LH/FSH (ratio)	2.80 ± 0.4	1.09 ± 0.2	<0.02
DHEA-S (μmol/L)	7.01 ± 2.5	5.50 ± 3.34	n.s
17-OH-P (nmol/L)	2.68 ± 0.46	1.46 ± 0.38	<0.05
A (nmol/L)	12.7 ± 1.2	6.08 ± 0.71	<0.02
T (nmol/L)	2.96 ± 0.2	0.85 ± 0.3	<0.005
E_2_ (pmol/L)	123 ± 26	104 ± 34	n.s.
SHBG (nmol/L)	24.6 ± 7.9	54.7 ± 13.6	<0.03
FAI (free androgen index)	12.0 ± 1.33	1.55 ± 0.6	<0.005
HOMA-ir	2.46 ± 0.42	1.45 ± 0.36	<0.05

**Figure 1:**
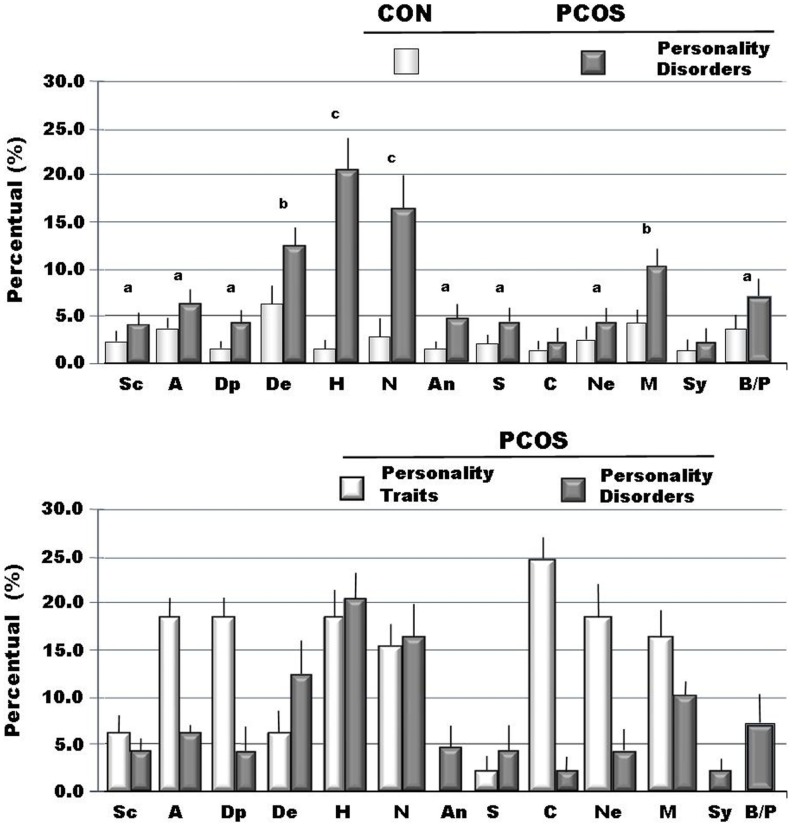
**The incidence of traits and of personality disorders (DSM-IV axis II) recorded through MCMI-III in personality scales (BR ≥ 75 is personality trait; BR ≥ 85 is disorder) (22)**. Legend: Sc, schizoid; A, avoidant; Dp, depressive; De, dependent; H, histrionic; N, narcissistic; An, antisocial; S, sadistic; C, compulsive; Ne, negativistic; M, masochistic; Sy, schizotypal; B, borderline; P, paranoid; ^a^*p* < 0.05 vs. Cont; ^b^*p* < 0.01vs. Cont; ^c^*p* < 0.005 vs. Cont.

**Figure 2:**
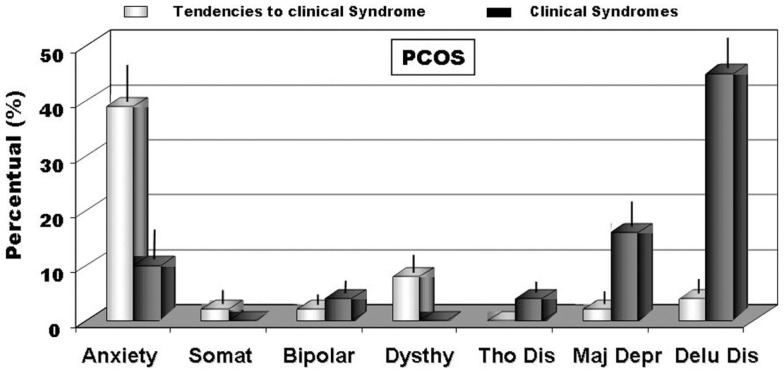
**The incidence of clinical disorders scales (DSM-IV axis I) (BR > 75) and of full blown clinical syndromes (BR > 85) recorded through MCMI-III (22)**. Legend: anxiety; Somat, somatoform; bipolar; Dysthy, dysthymia; Tho Dis, thought disorder; Major Dep, major depression; Delu Dis, delusional disorder.

**Figure 3:**
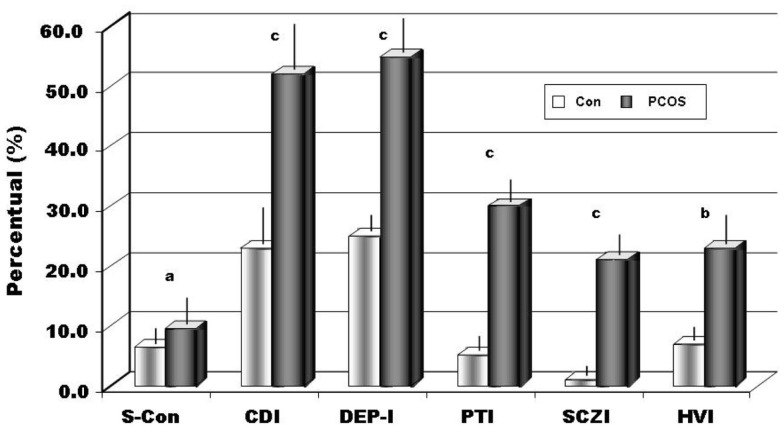
**The percentage in the sample of positive results to Rorschach indices (S-Con ≥ 8, CDI ≥ 4, DEP-I ≥ 5, PTI ≥ 3, SCZI ≥ 4, HVI ≥ 4 if *T* = 0)**. Legend: S-Con, suicide constellation; CDI, copy deficit index; DEP-I, depression index; PTI, perceptual-thinking index; SCZI, schizophrenia index; HVI, hypervigilance index; ^a^*p* < 0.05; ^b^*p* < 0.01;^c^*p* < 0.002.

When we analyzed personality disorders scales, by MCMI-III, in PCOS group, we observed that several diseases, diagnosed in according to DSM-IV axis II, reached percentage significantly higher than in controls. In Figure 1, in fact, we summarized the most important personality disorders in PCOS group compared with those observed in the control group. However, whether this increased incidence of personality disorders in patients affected by PCOS can be considered a link with clinical signs of hyperandrogenism remain at present unclear. In fact, the majority of studies showed a linkage between psychological disorders and major clinical PCOS symptoms, such as excessive body hairs, acne, infertility, or excessive body weight (4–6, 10, 13, 32, 33). Our results, in contrast, indicated that an intrinsic psychopathological characteristic of PCOS can be really consistent. According to our hypothesis, there are only three articles (17, 18, 34) that evidenced personality disorders in patients affected by PCOS, diagnosed by Diagnostic and Statistical Manual of Mental Disorders (DSM-IV). However, these latter studies recorded lower percentage of psychological disorders, when compared with our data, so suggesting that a heterogeneous expression of the same psychopathological diseases can exist in PCOS or it was assessed a heterogeneous pool of subjects affected by PCOS, therefore, further studies that consider the same Diagnostic Criteria for PCOS and a more numerous group of person are needed. When we considered the results of clinical syndrome scales, diagnosed according to DSM-IV axis I Criteria, we also found several clinical psychopathological disorders, as summarized in Figure 2. The most representative disorders were anxiety (about 10.0%) and depression (about 16.0%). These analyses, in fact, seem to confirm recent data obtained in PCOS patients (17), which showed a frequent finding of psychiatric disorders. Interesting to note that in our sample we didn’t observe neither alcohol addiction nor drug addiction or post-traumatic stress disorders. Because, we also observed different incidences (from 30.0% to about 70%) of psychopathological diseases, such as anxiety, mood, or thought disorders, using different psychometric test, it can be also suggested that different clinical expressions of psychopathological diseases can exist in PCOS. Also, other data, analyzed by different questionnaires, confirmed a prevalent expression of anxiety and depression, further suggesting that an elevated rate of mental illness in these hyperandrogenic patients (11, 14, 35). Again, Klipstein et al. (36) evidenced a rate of about 30.0% of bipolar disorder types I and II and Rassi (37) of about 11.0% of patients, respectively. Our data are in agreement with these latter results, so further evidencing that women with PCOS had a rate of mental illness several times higher (three to fivefold), when compared with normal population. However, the absence of rigorous diagnostic criteria of PCOS can not exclude an enrollment of heterogeneous groups of patients, so reducing the diagnostic capacity and, of consequent, the percentage of psychopathological diseases in the PCOS *per se*. Results of Rorschach test, obtained from our patients, are summarized in Table 2 and Figure 3. We used as control a group of 45 healthy Italian subjects already recorded in our database and gently provided by Zennaro (23). We used this trick because in Italy, at present, there are no any data about this pathology.

**Table 2:** **Comparison between PCOS and control groups using *t*-test for independent samples**.

Rorschach variables	PCOS group (*n* = 49)	Control group (*n* = 45)	*T*	*p*	Cohen’s *d*
				
	Mean ± SD	Mean ± SD			
Age	25.80 ± 4.27	24.80 ± 6.77			
R	21.59 ± 6.68	23.35 ± 8.170			
Lambda	0.606 ± 0.5576	0.5728 ± 0.4800			
FQM−	0.88 ± 1.3	1.18 ± 1.279			
FQS−	1.12 ± 1.39	1.63 ± 1.628			
FD	0.92 ± 0.91	1.35 ± 1.562			
XA%	69.94 ± 12.27	71.35 ± 12.949			
WDA%	75.06 ± 12.62	73.45 ± 11.964			
X + %	42.45 ± 13.24	47.58 ± 13.769	−1.777	0.079	−0.37
Xu%	27.45 ± 1.49	23.78 ± 9.983			
X − %	28.49 ± 11.99	27.78 ± 3.318			
S − %	16.57 ± 21.51	22.98 ± 22.273			
WSum6	23.55 ± 13.27	11.43 ± 10.77	4.759	0.000	1.003
WSumC	4.133 ± 2.345	3.338 ± 2.092			
EA	6.684 ± 3.556	7.888 ± 3.888			
ES	12.22 ± 6.28	11.68 ± 5.25			
D	-1.73 ± 2.09	-1.15 ± 1.46			
AdjD	-0.96 ± 1.65	-0.30 ± 1.159	−2.212	0.030	−0.464
Afr	0.445 ± 0.1605	0.488 ± 0.1281			
EGO index	0.42 ± 0.1685	0.4432 ± 0.1792			
EII	1.213 ± 1.0866	0.489 ± 1.1050	3.099	0.003	0.66
AG	0.20 ± 0.50	0.45 ± 0.815			
MOR	0.86 ± 1.17	2.05 ± 1.867	−3.515	0.001	−0.763
COP	0.51 ± 0.94	0.98 ± 1.121	−2.092	0.040	−0.454
GHR	2.12 ± 1.55	3.98 ± 1.790	−5.156	0.000	−1.110
PHR	3.47 ± 2.61	3.70 ± 2.44			
POP	4.20 ± 2.09	5.63 ± 1.917	−3.338	0.001	−0.712
Blends	5.67 ± 0.44	5.58 ± 3.727			
Color Sh. blends	1.41 ± 1.31	1.00 ± 1.320			
Shading Sh. blends	1.06 ± 1.34	0.33 ± 0.829	3.166	0.002	0.653
SumC′	3.08 ± 2.26	2.53 ± 1.894			
SumT	1.43 ± 1.34	0.75 ± 0.776	2.986	0.004	0.621
SumV	1.65 ± 1.70	0.70 ± 0.966	3.319	0.001	0.686
SumY	2.53 ± 1.95	1.88 ± 2.174			
Sum shading	8.69 ± 4.85	5.85 ± 4.123	2.990	0.004	0.631
Fr + Rf	0.71 ± 0.87	0.83 ± 1.130			
IsoIndex	0.226 ± 0.1222	21.28 ± 12.649			
Sum6	7.02 ± 3.53	3.68 ± 2.99	4.842	0.000	1.021
Intell	2.59 ± 2.04	2.85 ± 2.842			
An + Xy	1.96 ± 0.3	0.90 ± 1.215	2.783	0.007	0.576
Schizo index	2.88 ± 1.48	2.68 ± 1.70			
PTI	1.80 ± 1.41	1.63 ± 1.59			
HVI 2–8	2.29 ± 1.53	3.23 ± 2.057	−2.398	0.019	−0.518
DEP-I	4.73 ± 1.22	4.25 ± 1.214	1.869	0.065	0.394
CDI	3.33 ± 1.3	2.90 ± 1.128			
S-Con	5.88 ± 1.18	5.40 ± 1.736			

Using a statistical comparison (Student’s *t*-test for independent groups) between the samples of patients and the control group, we have evidenced significant differences among several variables, as showed in Table 2. Calculating Cohen’s *d*, the effect size (statistically significant >0.6) underlined a high effect for 10 of these variables: WSum6, AdjD, ego impairment index (EII), MOR, Popular, Shading Blends, SumT, SumV, Sum Shading e Sum6. Our data show an important deficit in cognitive functions (MEDIATION and IDEATION clusters). Cohen’s *d* underlines as “high effects” (≥0.6) of the following variables: X + %, WSum6, AdjD, COP, GHR, Popular, SumV, SumY, Sum Shading, and Sum6. The following variables, WSum6, SumV, Sum Shading, Sum6, are significantly higher. The variables, X + %, COP, GHR, Popular, were significantly lower, while AdjD was actually negative. All these data evidenced that in subjects with PCOS exists a massive reality distortion (XA%, X − %, X + %, EII, PTI index, and SCZI index) with incoherent and disorganized thoughts (Sum6 and Wsum6), failures in the ability to judge, bizarre, or magical ideations and with interpersonal communication difficulties. PTI was positive for 30.6% of patients as well as SCZI for 20.4% of our sample, therefore, we can underline as “*daily life*” is really difficult for the majority of our patients affected by PCOS, because misperception leads to behavior often inappropriate in social life. Again, a large proportion (63.3%) of patients (X − % > 0.25) were displayed serious perceptual inaccuracies, stressing therefore how the reality was not adequately perceived because emotions distorted it (X − %, X + %). Again, 42.9% of subjects (Xu% = 0.2) were anti-conformism, displaying a tendency to originality, but 53.1% (Populars ≤ 4) rejected conventional models, so adopting behaviors not socially adequate and/or accepted. Ninety-two percent of subjects with PCOS (X + % <0.70) showed a perception of the situations and words in an unusual manner. About four (4.1) % (Populars ≥ 8) of our sample was exaggerated in conformism. These latter patients usually need a positive social judgment and they need the approval of other people. In our sample, 26.5% (lambda < 0.30) had an inadequate control over emotions that interfere with logical functions. EII showed serious impairment in 38.8% of the sample, moderate in 18.4%, average in 8.2%, and slight in 24.5% (X − %, Sum6, WSum6 and EII higher, XA%, WDA%, X + % lower). Only 10.0% of women analyzed, had a normal, non-pathological values. According all these results, we evidenced that our patients had really difficulties in organizing the stimulus when they face the reality, so forcing them to use more psychic energy than needed (WDA < XA%). However, in our study about 12.2% of patients (12 subjects) ignored the complexity of the reality and tended to simplistic situations and interpersonal relationships. Again, 40.8% were very self-involved (Egoindex ≥ 0.45) and about 30% built up their own self-esteem, by considering themselves better than others (Fr + Rf = 1). This focus on self could be detrimental in the interpersonal relationship and could contribute to reality distortion. Our data seem to confirm Willmott’s results (38) that evidenced as women with PCOS exhibit a lack of control over their own lives’ events. Considering the CDI, we found it positive for 53.1% of the patients (vs. 29% of the controls), revealing a deficit in coping abilities, and consequently interpersonal relationships were superficial and rarely maintained. Also, social skills appeared to be reduced (CDI, EA) and interpersonal relationships were perceived as unsatisfying. About thirty (30.6) % of PCOS sample had a damaged and negative interpersonal relationship representation (PHR higher). Interactions of personal situations were perceived as not cooperative, but in a hostile manner. All of this creates, in our patients, a state of chronic stress, which was reflected in a cognitive slippage. More than 50% of our patients resulted depressed (DEP-I positive) and with a percentage significantly higher than observed in controls (55.1 vs. 25.0% controls). Unexpectedly, 8.1% of patients with PCOS (vs. 6% of the controls) were at risk for suicide (S-Con positive, MOR = 2, FD > 2, Shadings). These latter results were in agreement with previous studies that underlined how this psychopathological tendency was more frequently observed in women with PCOS (35). However, at present, whether suicide can be cause or effect of high level of discomfort induced by clinical or psychological signs, or both, remains still unknown. In expected manner, several authors (39, 40) reported a high prevalence of negative self image and poor body image in subjects with PCOS. Also, in our PCOS group, with respect of controls, we evidenced a significant higher, about 18.0%, a self image damaged (MOR = 2). Again, about 45% of patients had an exaggerated introspective behavior (FD ≥ 2) that it could became a pathological self criticism and a ruminative thinking. This point of view is important, because women with PCOS had a negative and pessimistic view of themselves; therefore, they had constant need of reassurance about their own value and had serious problems of establishing intimate relationship (18.4% Fr + Rf ≥ 2). In our patients, we recorded about 25% of the samples (vs. 7% of healthy and normal subjects) constantly in alert (HVI) and consequently worried, therefore, this mood justifies how the majority part of the subjects examined, about 64% of PCOS, lived with a constant state of anxiety (SumY ≥ 2). It was interesting to underline that similar results were obtained both in depressed patients and in subjects with juvenile diabetes mellitus type I (30). And again, it was suggested that the observation of Monzani et al. (41) who emphasized how in subjects affected by PCOS scores of somatization, anxiety, and depression variables reached higher values when compared with normal people. At the same time, these authors interestingly did not observe any relevant psychopathological disorders, but differently noticed that the subjects tend to hidden and/or clear behaviors typical of the opposite gender. Moreover, the same women tended to conform themselves to social standards, so probably reducing the perception of their “diversity.” Our data, therefore, seem to confirm these results that report how ill women have altered body self-perception, sexual intimacy, sexual dissatisfaction, and the strong opinion that their partners’ sex life is as unsatisfactory as their own, so indicating that PCOS is perceived as a real disease (42).

About 60% of PCOS women experienced painful emotions and high sense of insecurity, confusion, and ambivalence in relation to emotions (Color Shading Blends > 1 in 69.4% and Shading Blends > 1 in 61.2%), showed a low self-esteem (36.7% with Egoindex < 0.32), and feel anxious, tense, nervous, and irritable (more than 71.0% showed FQS-higher). All these data evidenced that these women did not tolerate frustration and they were impulsive (D < 0). About 70.0% was feared to affective stimuli and avoid them (Afr lower) and experienced discomfort with introspection, because feelings of inferiority and depressed feelings (SumV ≥ 1). We observed that many of the samples (69.4%) did not use intellectualization (Intell > 3) as a defense mechanism against affects, but tended to avoid (Afr) situations where they were generated. Since several patients affected by PCOS tended to isolate themselves, to refuse interpersonal relationships, to present social phobia, or tended to be worried and keep at a certain emotional distance from others, it can be concluded that these subject are not very interested in interpersonal relationship. However, our results, in different manner, evidenced that about 60% of patients tended to actively research contact and interpersonal relationships (Isoindex < 0.25). Because, we studied a group of young patients, with the majority of them in a period of university study, where the interpersonal contacts are very high, we cannot exclude that we have enrolled a group of patients with different social or cultural impact.

## Conclusion

Our results clearly highlight that in our patients, affected by PCOS, exist mood and severe thought disorders with perceptual distortion in about half of subjects. In this hyperandrogenic syndrome, women have elevated dysphoric feelings, chronic emotional stress, and several difficulties in social skills and daily life. In our group of patients, to attenuate an important painful emotional overload, many avoid interpersonal relationships and emotional inputs. Again, intimate relationships can often cause fear and frustration. All these conditions suggest that the characteristics of psychological suffering in PCOS can constitute a distinguishing element, which, for some aspects, appears to be so represented and shared by the sample as to be promoted to a proper diagnostic indicator. We also underline that the knowledge of these psychopathologic aspects may ameliorate the doctor–patient interaction and relationship with a desirable improvement on the medical examination, sensibility, and probably reaching greater adherence to diagnostic and medical therapy.

## Conflict of Interest Statement

The authors declare that the research was conducted in the absence of any commercial or financial relationships that could be construed as a potential conflict of interest.

## References

[1] Rosenfield RL Ghai K Ehrmann DA Barnes RB. Diagnosis of the polycystic ovary syndrome in adolescence: comparison of adolescent and adult hyperandrogenism. J Pediatr Endocrinol Metab (2000) 13(Suppl 5):1285–9. 11117671

[2] Azziz R Carmina E Dewailly D Diamanti-Kandarakis E Escobar-Morreale HF Futterweit W Task force on the phenotype of the polycystic ovary syndrome of the androgen excess and PCOS society. The androgen excess and PCOS society criteria for the polycystic ovary syndrome: the complete task force report. Fertil Steril (2009) 91:456–88. 10.1016/j.fertnstert.2008.06.035 18950759

[3] Diamanti-Kandarakis E Dunaif A. Insulin resistance and polycystic ovary syndrome revisited: an update on mechanisms and implications. Endocr Rev (2012) 33(6):1–29. 10.1210/er.2011-1034 23065822 PMC5393155

[4] Barnard L Ferriday D Guenther N Strauss B Balen AH Dye L. Quality of life and psychological well being in polycystic ovary syndrome. Hum Reprod (2007) 22(8):2279–86. 10.1093/humrep/dem108 17537782

[5] Coffey S Mason H. The effect of polycystic ovary syndrome on health-related quality of life. Gynecol Endocrinol (2003) 17(5):379–86. 10.1080/09513590312331290268 14710585

[6] Li Y Li Y Yu Ng EH Stener-Victorin E Hou L Wu T Polycystic ovary syndrome is associated with negatively variable impacts on domains of health-related quality of life: evidence from meta-analysis. Fertil Steril (2011) 96(2):452–8. 10.1016/j.fertnstert.2011.05.072 21703610

[7] Hahn S Janssen OE Tan S Pleger K Mann K Schelowski M Clinical and psychological correlates of quality of life in polycystic ovary syndrome. Eur J Endocrinol (2005) 153:853–60. 10.1530/eje.1.02024 16322391

[8] Barry JA Kuczmierczyk AR Hardiman PJ. Anxiety and depression in polycystic ovary syndrome: a systematic review and meta-analysis. Hum Reprod (2011) 26(9):2442–51. 10.1093/humrep/der197 21725075

[9] Deeks AA Gibson-Helm ME Paul E Teede HJ. Is having polycystic ovary syndrome a predictor of poor psychological function including anxiety and depression? Hum Reprod (2011) 26(6):1399–407. 10.1093/humrep/der071 21436137

[10] Annagur BB Tazegul A Uguz F Kerimoglu OS Tekinarslan E Celik C. Biological correlates of major depression and generalized anxiety disorder in women with polycystic ovary syndrome. J Psychosom Res (2013) 74:244–7. 10.1016/j.jpsychores.2013.01.002 23438716

[11] Sills ES Perloe M Tucker MJ Kaplan CR Genton MG Schattman GL. Diagnostic and treatment characteristics of polycystic ovary syndrome: descriptive measurements of patient perception and awareness from 657 confidential self-reports. BMC Womens Health (2001) 1(1):3. 10.1186/1472-6874-1-1 11545683 PMC55341

[12] Trent ME Rich M Austin F Bryn S Gordon CM. Quality of life in adolescent girls with polycystic ovary syndrome. Arch Pediatr Adolesc Med (2002) 156(6):556–60. 10.1001/archpedi.156.6.556 12038887

[13] Dokras A Clifton S Futterweit W Wild R. Increased risk for abnormal depression scores in women with polycystic ovary syndrome. Obstet Gynecol (2011) 117(1):145–52. 10.1097/AOG.0b013e318202b0a4 21173657

[14] Jedel E Waern M Gustafson D Landern M Eriksson E Holm G Anxiety and depression symptoms in women with polycystic ovary syndrome compared with controls matched for body mass index. Hum Reprod (2010) 25(2):450–6. 10.1093/humrep/dep384 19933236

[15] Dokras A Clifton S Futterweit W Wild R. Increased prevalence of anxiety symptoms in women with polycystic ovary syndrome: systematic review and meta-analysis. Fertil Steril (2012) 97(1):225–30. 10.1016/j.fertnstert.2011.10.022 22127370

[16] Stovall DW Scriver JL Clayton AH Williams CD Pastore LM. Sexual function in women with polycystic ovary syndrome. J Sex Med (2012) 9:224–30. 10.1111/j.1743-6109.2011.02539.x 22082203 PMC3643123

[17] Sahingöz M Uguz F Gezginc K Korucu DG. Axis I and axis II diagnoses in women with PCOS. Gen Hosp Psychiatry (2013) 35(5):508–11. 10.1016/j.genhosppsych.2013.04.003 23726743

[18] Roepke S Ziegenhorn A Kronsbein J Merkl A Bahri S Lange J. Incidence of polycystic ovaries and androgen serum levels in women with borderline personality disorder. J Psychiatr Res (2010) 44:847–52. 10.1016/j.jpsychires.2010.01.007 20149393

[19] The Rotterdam Consensus ESHRE/ASRM: ESHRE/ASRM Rotterdam Consensus Meeting. Revised 2003 consensus on diagnostic criteria and long-term health risks related to polycystic ovary syndrome (PCOS). Hum Reprod (2004) 19:41–7 10.1093/humrep/deh098 14688154

[20] Fulghesu AM Angioni S Frau E Belosi C Apa R Mioni R Ultrasound in polycystic ovary syndrome – the measuring of ovarian stroma and relationship with circulating androgens: results of a multicentric study. Hum Reprod (2007) 22(9):2501–8. 10.1093/humrep/dem202 17635847

[21] Matsuda M DeFronzo RA. Insulin sensitivity indices obtained from oral glucose tolerance testing: comparison with the euglycemic insulin clamp. Diabetes Care (1999) 22:1462–70. 10.2337/diacare.22.9.1462 10480510

[22] Millon T Davis R Millon C MCMI III, Manual. 2nd ed Minneapolis, MN: NCS Inc. (1997).

[23] Zennaro A Ferracuti S Lang M Sanavio E L’adattamento italiano del MCMI-III. Studi di validazione [MCMI-III Italian Adaptation]. Firenze: Organizzazioni Speciali (2008).

[24] Exner J The Rorschach Comprehensive System: Volume 1: Basic Foundations. 2th ed New York, NY: Wiley (1986).

[25] Exner J The Rorschach Comprehensive System: Volume 1: Basic Foundations. 2th ed New York, NY: Wiley (1995).

[26] Exner JE Eldberg P The Rorschach: A Comprehensive System: Volume 2: Advanced Interpretation. 3rd ed New York, NY: Wiley (2005).

[27] Lis A Zennaro A Salcuni S Parolin L Mazzeschi C Il Rorschach secondo il Sistema Comprensivo di Exner. Milano: Raffallo Cortina Editore (2007).

[28] Exner JE Andronikof-Sanglade A. Roschach changes following brief and short-term therapy. J Pers Assess (1992) 50(1):59–71. 10.1207/s15327752jpa5901_6 1512680

[29] Bihlar B Carlsson AM. An exploratory study of agreement between therapists’ goals and patients’ problems revealed by the Rorschach. Psychother Res (2000) 10(2):196–214. 10.1080/10503307.2000.9721973 22239697

[30] Sultan S Jebrane A Heurtier-Hartemann A. Rorschach variables related to blood glucose control in insulin-dependent diabetes patients. J Pers Assess (2002) 79(1):122–41. 10.1207/S15327752JPA7901_08 12227663

[31] Mioni R Chiarelli S Xamin N Zuliani L Granzotto M Mozzanega B Evidence for the presence of glucose transporter 4 in the endometrium and its regulation in polycystic ovary syndrome patients. J Clin Endocrinol Metab (2004) 89(8):4089–96. 10.1210/jc.2003-032028 15292352

[32] Sonino N Fava GA Mani E Belludo P Boscaro M Quality of life of hirsute women. Postgrad Med J (1993) 69:186–9 10.1136/pgmj.69.809.186 8497431 PMC2399746

[33] Ekback M Wijma K Benzein E. “It’s always on my mind”: women experiences of their bodies when living with hirsutism. Health Care Women Int (2009) 30:358–72. 10.1080/07399330902785133 19350434

[34] Marsh CA Berent-Spillson AB Love T Persad CC Pop-Busui R Zubieta JK Functional neuroimaging of emotional processing in women with polycystic ovary syndrome: a case-control pilot study. Fertil Steril (2013) 100(1):200–7. 10.1016/j.fertnstert.2013.02.054 23557757 PMC3900232

[35] Mansson M Holte J Landin-Wilhelmsen K Dahigren E Johansson A Landen M. Women with polycystic ovary syndrome are often depressed or anxious – a case control study. Psychoneuroendocrinology (2008) 33:1132–8. 10.1016/j.psyneuen.2008.06.003 18672334

[36] Klipstein KG Goldberg JF. Screening for bipolar disorder in women with polycystic ovary syndrome. J Affect Disord (2006) 91(2–3):205–9. 10.1016/j.jad.2006.01.011 16487597

[37] Rassi A Veras AB Dos Reis M Pastore DL Bruno LM Bruno RV Prevalence of psychiatric disorders in. Compr Psychiatry (2010) 51:599–602. 10.1016/j.comppsych.2010.02.009 20965306

[38] Willmott J The experiences of women with polycystic ovary syndrome. Fem Psychol (1999) 10(1):107–16 10.1177/0959353500010001013

[39] Mansson M Norstrom K Holte J Landin-Wilhelmsen K Dahlgren E Landen M. Sexuality and psychological wellbeing in women with polycystic ovary syndrome compared with healthy controls. Eur J Obstet Gynecol Reprod Biol (2011) 155:161–5. 10.1016/j.ejogrb.2010.12.012 21232840

[40] Bazarganipour F Ziaei S Montazeri A Foroozanfard F Kazemnejad A Faghihzadeh S. Psychological investigation in patients with polycystic ovary syndrome. Health Qual Life Outcomes (2013) 11:141. 10.1186/1477-7525-11-141 23947827 PMC3751454

[41] Monzani F Pucci E Caraccio N Bagnolesi A Molli D Fenu A Correlati psicologici and psicopatologici nella sindrome della micropolicistosi ovarica. Med Psicosom (1994) 39:225–36.

[42] Elsenbruch S Hahn S Kowalsky D Offner A Schedlowski M Mann K Quality of life, psychosocial well-being, and sexual satisfaction in women with polycystic ovary syndrome. J Clin Endocrinol Metab (2003) 88(12):5801–7. 10.1210/jc.2003-030562 14671172

